# Quantifying Tactile Perception of Fabrics Using Both Frictional and Acoustic Methods

**DOI:** 10.1007/s11249-026-02107-2

**Published:** 2026-01-14

**Authors:** Laure Kyriazis, Tugce Caykara, Daniel Ingo Hefft, Alberto Martinez, Zhenyu Jason Zhang

**Affiliations:** 1 School of Chemical Engineering, University of Birmingham, Edgbaston, Birmingham, UK; 2 Procter & Gamble, Brussels Innovation Center, Strombeek-Bever, Belgium

**Keywords:** Tactile perception, Skin tribology, Acoustic emission

## Abstract

Skin friction underpins tactile perception of formulated products, such as cosmetics, fabric softeners, and surface coatings, that are designed to deliver satisfactory tactile sensory properties. Establishing the correlation between the tribological properties of skin contacts and tactile perception is therefore critical. We have developed a novel approach to acquire the acoustic emission (AE) signal generated by human finger sliding against fabric and non-fabric substrates, whilst capturing its frictional characteristics with a force plate. Principal Component Analysis was deployed to construct clusters formed for each material based on the sensory evaluation, and to establish the correlation between frictional and acoustic emission data. Our results show that the planar solid substrates could be discriminated solely based on the friction results, whilst the fabric materials were not significantly discriminated by the Coefficient of Friction values. However, AE was able to differentiate the fabric substrates successfully, and therefore evidences a promising potential to provide complementary information on tactile perception whilst being versatile enough as a standalone method.

## Introduction

Tactile perception of human is determined by both physical and psychological processes [1], which is challenging to understand fully. The presence of various mechano-sensory cells (Merkel cells, Meissner corpuscles, Ruffini endings, and Pacinian corpuscles) at different depth profiles of human skin allows human to sense frictional force, amongst other stimuli involved in the tactile perception of objects [2]. When a finger slides against a solid substrate, the sensory cells would produce signals, according to the compressive and tensile deformations, that are transmitted to the brain, leading to the tactile perception of different properties of the materials in contact.

Tactile perception is determined by multiple factors, of which the most important one is the finger friction [3], conventionally evaluated by a friction rig. Such laboratory-based skin friction measurements provide an objective and quantitative method to evaluate the physical factors that influence tactile perception, minimising the test subjectivity of the sensory panel. From a tribological perspective, skin friction is governed by the properties of the solid substrate (physico-chemical and topological characteristics), human skin (physiological conditions such as hydration and sebum levels [4]), and contact parameters (e.g. contact area, normal load, velocity, and Young’s moduli).

Skin friction measurement methods can be classified into two categories: passive tests using a probe against the skin and active touch experiments [5]. Adams and colleagues investigated the frictional characteristics between spherical probes (smooth glass and polypropylene) and human forearm in dry, damp, and wet states, and reported that the increased contact area, with the presence of water, was the main factor causing the increased friction in a wet state [6]. Ramalho and co-workers designed a hand-held probe to measure the Coefficient of Friction (COF) between different types of fabrics in a rotating contact with the forearm and palm of the participants, and reported a similar ranking with the friction and some sensory properties (slipperiness and smoothness) assessed with a questionnaire [7]. Derler et al. compared the sliding friction of human skin and artificial skin in contact with fabrics to identify a preferred skin surrogate candidate [8]. As an alternative, a biomimetic finger was developed to replicate human touch. For instance, a haptic measurement system was used by Skedung and co-workers to study the tactile perception of surface coatings commonly used in furnishing products. Such setup measures 15 different precepts describing texture, friction, compliance, or temperature of the samples probed by the artificial finger [9]. Tactile perception of topical formulations was studied by Skedung et al. using finger friction measurements on an artificial skin surface with an even film of model formulations—they were able to detect small changes due to the physical properties of such organic thin film [10]. In one of the recent studies, Zhang and colleagues attempted to establish the connection between skin friction and brain response, and reported that the deformation friction and vibration amplitude of skin increase with an increasing surface roughness [11].

Masen chose to study the reciprocating movement of an index finger on stainless steel disks; therefore, an active touch experiment for the ‘inherent simplicity’ [12]. Such measurement highlights the importance of the skin deformation component in the Coefficient of Friction measured, especially in the wet state. Although tribological measurements whereby probes are rubbed against the skin allow for better control of the tribological contact parameters such as sliding velocity and applied normal load, human active touch experiment is preferred for a better understanding of finger friction and the correlated tactile perception.

When it comes to the tactile perception of fabrics, surface and frictional properties are determined by a combination of parameters such as fibre materials [13], fabric construction, surface finishing [14], and history of surface treatment [15]. The considerable number of possible combinations of the physical properties of fabrics adds an additional layer of complexity in understanding the tactile perception of textiles since they strongly influence the tactile properties of the different fabrics. For example, Gerhardt and colleagues measured skin-textile friction using three types of fabrics, bamboo viscose (sateen 4/1 structure), polytetrafluoroethylene (PFTE) (twill ½ S structure), and 50% cotton/50% polyester (plain 1/1 structure) when recruiting 60 panellists—the difference in COF between the fabrics was observed [13]. Investigating the role of skin friction on fabrics is of key importance to understand the effects of tactile perception and consumer preferences, providing an objective and quantitative evaluation whilst reducing the time and cost associated with sensory panels.

However, conventional tribological measurement of using a mechanical device is not suitable for skin friction from both measurement and practicality perspectives. Standard force-plate apparatus measurements are limited by the size of the measurement stage and the frictional axis (where the alignment of the sliding probe and substrate is of paramount importance for friction signal accuracy); therefore, large contacting substrates such as human skin and different types of sliding contacts such as rotational sliding cannot be investigated. Additionally, for contacts between skin and deformable substrates, such as fabrics, quantification of surface friction solely would have limitations because the deformability of the contacting materials is not really considered but has a great impact on the effective contact area and friction. In this context, the use of an acoustic emission (AE) sensor could be promising in quantifying the human skin tribological contacts.

Acoustic emission refers to the measurement of the high-frequency stress waves emitted by the rapid release of energy of a material during a mechanical process (e.g. friction, crack propagation, impact), following which strain transmits rapidly within the material. Such events take place in the ultrasound region that is beyond 20 kHz. The high-frequency transient elastic waves propagate within the material and can be detected by a piezoelectric sensor placed on the surface of the substrate investigated. The pressure fluctuation signal is converted to an electric signal and amplified to convey the signal to the data acquisition unit. Acquisition of the AE signal is therefore a non-invasive and non-destructive evaluation technique that allows the measurement of the real-time transient energy dissipation of a process, without the need to rely on the introduction of reference waves (energy input) as it would be the case using techniques such as pitch-and-catch and pulse-echo [16, 17].

Acoustic techniques such as AE are widely used in civil engineering [18] and chemical engineering [19–21], and have most recently raised interest in the tribology field [22–27]. Wear and friction of hard sliding contacts, such as steel-on-steel, are the most extensively investigated ones. Reddyhoff and co-workers demonstrated the ability of AE in investigating the dry sliding contact of steel-on-steel ball-on-cylinder, identifying the characteristic acoustic emission frequencies that reflect the friction or wear of the contact [22]. Benabdallah and Aguilar studied a similar AE system and reported a satisfactory correlation between the Coefficient of Friction and the Root Mean Square (RMS) of the AE signal [17]. On the other hand, investigation of the AE signal generated upon contact of soft materials, especially tactile perception, is less understood. Dearn and colleagues used AE to study a simulated metal-on-polymer joint replacement articulation and predicted the Coefficient of Friction using a non-linear autoregressive neural network [25]. Stroking contact between skin and dry hair was investigated using AE, which enabled McMullen and colleagues to evaluate hair surface properties [28]. AE was measured upon rubbing and tapping contacts of skin and tongue surfaces by van Aken to link them with tactile perception [29]. This work showed the dependence of rubbing and adhesive contact with the finger for varying surface textures and demonstrated the great potential of AE for in vivo measurements. In the most recent work, Reddyhoff and colleagues applied machine learning (ML) and a data-driven method to AE data acquired from a lubricated sliding contact to predict the complete Stribeck curve [30] and other tribological characteristics [31], which demonstrates the advantage of AE, as a methodology, in generating a large volume of results for ML.

Limited attempts have been made to investigate the acoustic signal generated by touching fabrics, though. Measuring the audible sound wave generated upon friction of fabrics was pioneered by Cooper with a microphone [32]. The frequency-domain acoustic profiles were established for frequencies up to 14 kHz, where magnitude differences were observed for different types of fabrics. The total acoustic signal emitted was correlated with the fabric roughness. Leclinche and co-workers investigated the sensory perception of frictional sounds of fabrics [33] and how the audible acoustic signal (measured with a microphone for frequencies up to 11 kHz) correlated with mechanical properties [34]. Valuable information could be obtained with the acquisition of high-frequency acoustic friction response of textiles, which to our knowledge has not yet been studied in the literature for frequencies above the audible range.

In the present work, we developed a method to acquire both frictional force and acoustic emission signal generated by human finger touching a range of solid substrates and fabrics in situ. This new approach was subsequently used to evaluate the sensory properties of varying materials, with the aim of correlating the quantitative results obtained from friction and acoustic emission experiments with tactile perception.

## Materials and Methods

### Materials

**Fig. 1:**
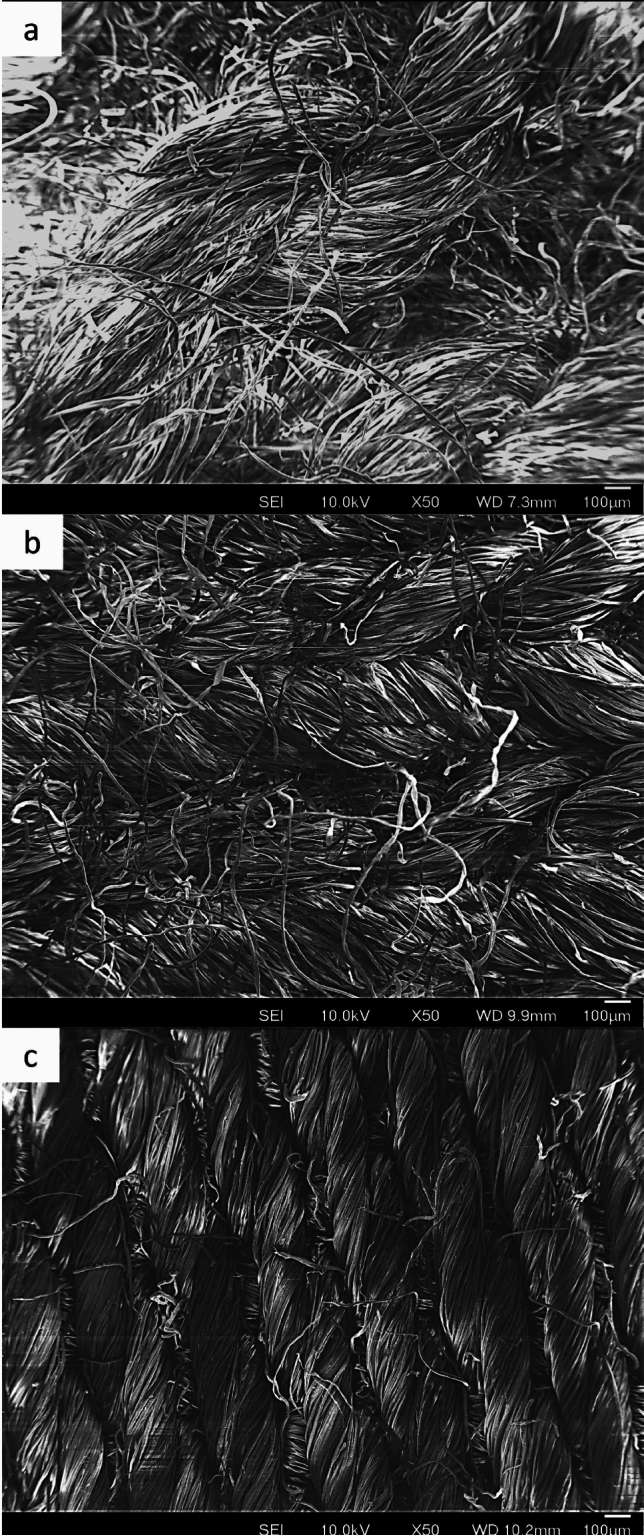
SEM micrographs of fabrics investigated: **a** cotton terry, **b** polycotton knit, and **c** cotton woven

A selection of six solid materials with distinctively different physical and surface characteristics were investigated in the present work, including three types of fabrics: cotton terry (Calderon, USA), cotton woven, and 50–50 polyester-cotton jersey knit (TestFabrics, USA), and three planar substrates: glazed ceramic (Emaillerie Belge, Belgium), polyethylene terephthalate (PET) film (Mylar, DuPont Teijin Films, UK), and Polytetrafluoroethylene (PTFE) tape (Fortspang PTFE-coated tape). The three types of fabrics investigated differ in the fibres used (single-sourced staple fibres (cotton) or a blend (polyester-cotton blend)), structure (woven, knit, or terry), hairiness (terry fabrics have loops sticking out of the surface), and porosity of the structure. Any pre-treatment during the manufacturing process was eliminated from all fabrics with multiple wash cycles in washing machines (with detergent and water only cycles). All skin friction measurements were performed in a temperature (25°C) and relative humidity (40% RH) controlled room, where the samples were left to equilibrate prior to each of the experiments and sensory evaluation. Scanning Electron Microscopy (SEM) images of the fabrics present the top views of the fabric surfaces investigated (Fig. 1).

### Measurement and Sensory Panel

A total of eight human subjects, aged between 23 and 33 years old, were recruited for the finger friction and sensory experiments (4 males and 4 females). Consent from the participants was given prior to commencing the measurements. The subjects were also informed that they could withdraw from the study at any time should they wish to do so. The work received approval (ERN-1748-Oct2023) from the University of Birmingham Research Ethics panel.

Every participant washed their hands thoroughly with hand soap and dried them with a paper towel, prior to starting the sensory evaluation as well as prior to each new sample measured with finger friction.

### Sensory Evaluation

**Fig. 2:**
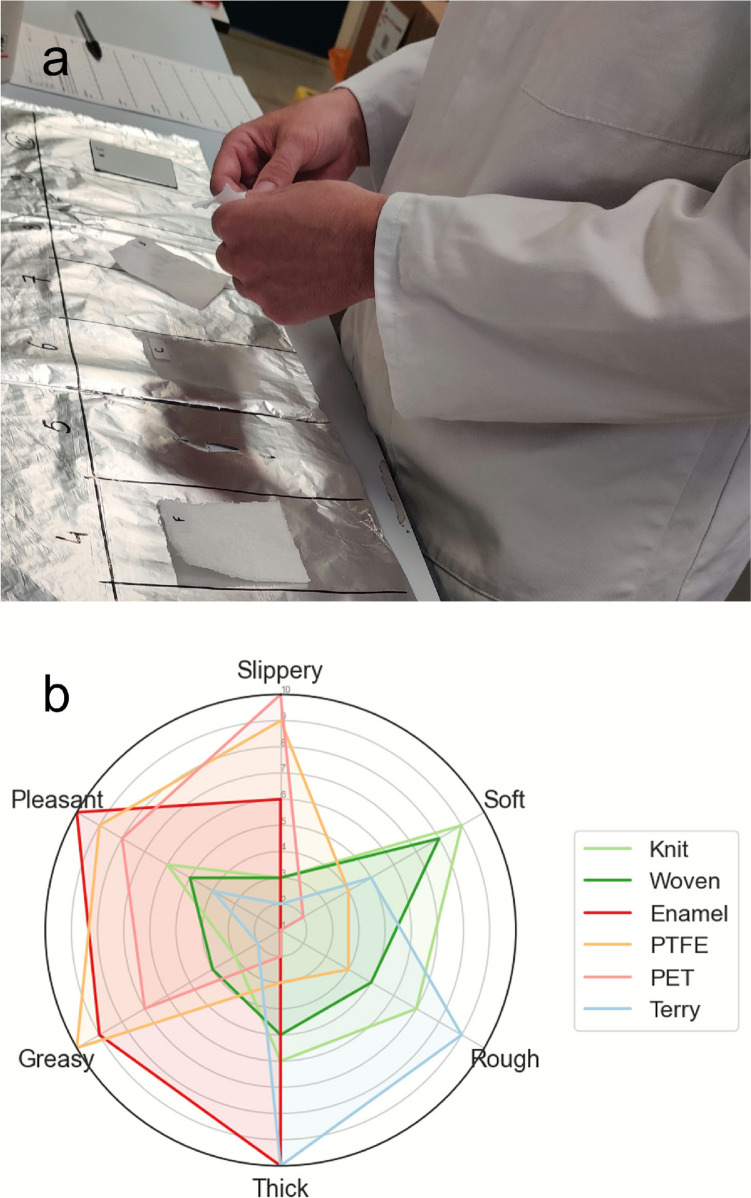
**a** Sensory evaluation setup and **b** representative response to questionnaire used in the present work

A questionnaire was provided to each panellist prior to the experiment for them to assess the different sensory characteristics (stickiness, softness, roughness, thickness, greasiness, and overall feeling) to be evaluated, see sensory evaluation in Fig. 2. The tactile sensory characteristics investigated in this study were adapted from a previous tactile perception study [35], but only included the most relevant ones possible, as well as the simplest for untrained panellists to identify.

A code letter (from A to F) was randomly assigned to each of the specimens and placed next to the interval rating scale (ranging from 1 to 10). For each of the 6 characteristics evaluated, participants were asked to compare the attributes for all the samples and place them accordingly on the scale, having at least one sample placed on each of the extreme grades (1 and 10 being the lowest and highest grades, respectively). The results of the sensory test were subsequently captured on the corresponding scale of the sensory evaluation questionnaire once the subject was satisfied with their current rating.

A representative response to the questionnaire, captured in a radar chart, is shown in Fig. 2, where each axis represents the parameters evaluated. Characteristic profiles of each of the materials evaluated by the participant can then be visualised.

### Finger Friction Experiments

**Fig. 3:**
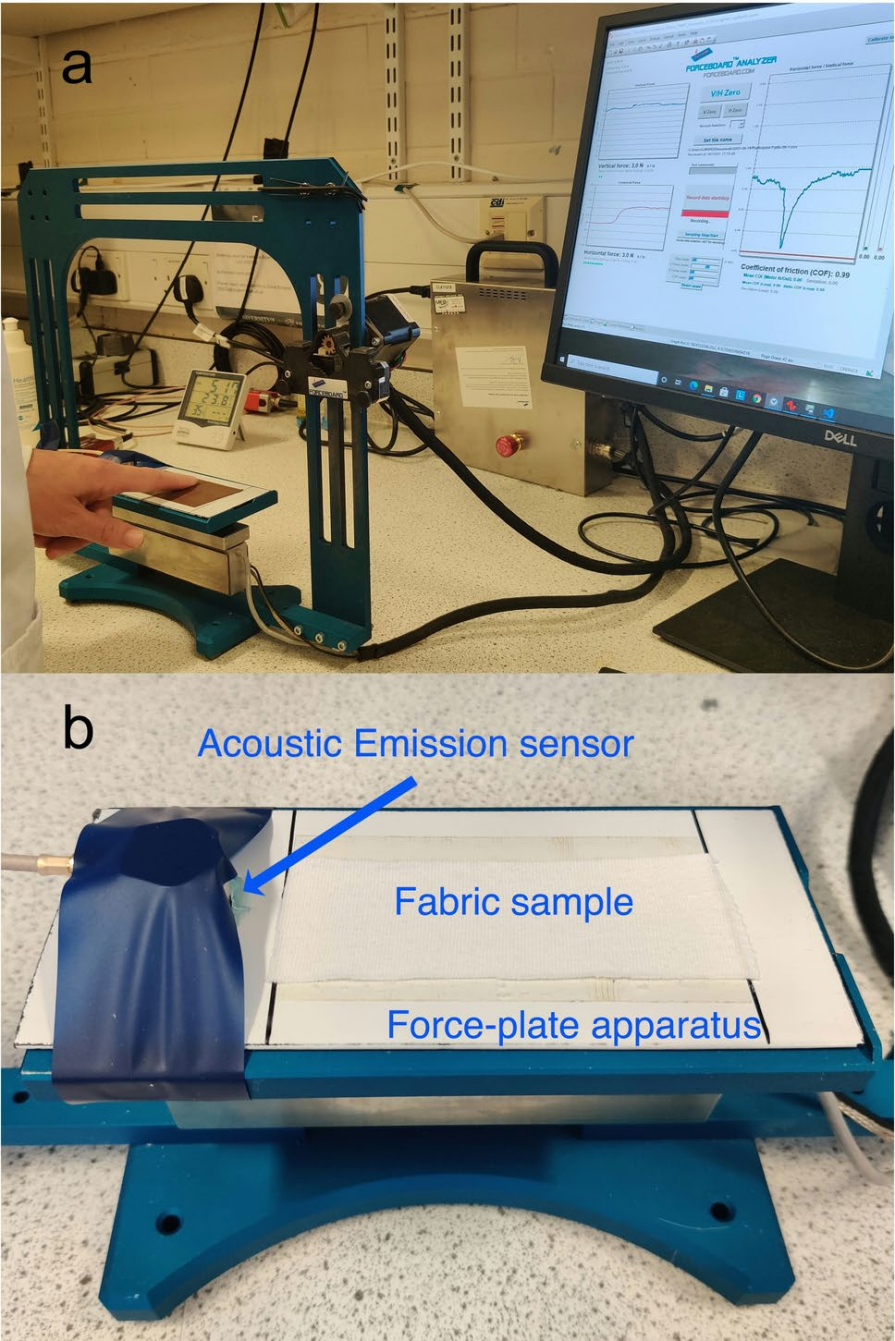
**a** Experimental setup for finger friction with real-time force measurement visualisation, and **b** a close-up view of the measurement stage on which the AE sensor is fixed by a blue tape

The friction of the human finger in reciprocating sliding contact with the substrates was measured by a force-plate apparatus (ForceBoard, Sweden) and an acoustic emission sensor (Vallen, Germany), simultaneously. The experimental setup is shown in Fig. 3.

For each experiment, the participant was asked to place the index finger of their dominant hand on the object and act with a continuous and reciprocal movement from left to right, at a steady velocity and a target-applied load (2 N, 3 N, or 4 N). The applied load was actively controlled by the experimenters, thanks to real-time prompting of the force measured on the software. The experimenters were given some time prior to the measurements to train themselves in applying the normal force as closely as possible to the target normal load for every sample. The specimens were evaluated one at a time, in randomised order. For each sample and load applied investigated, 3 repeats of 30 sec recordings were acquired for each of the participants.

#### Friction Rig

A force-plate apparatus (ForceBoard System, ForceBoard, Sweden) was used to measure the vertical forces and horizontal forces acting in real time on the sample positioned on the board during the sliding movements of the human finger, at a sampling rate of 40 Hz. A customised Python code was used to calculate the Coefficient of Friction by dividing the friction forces (horizontal direction) with the normal forces applied in the vertical direction over time, as well as to perform further data analysis.

**Fig. 4:**
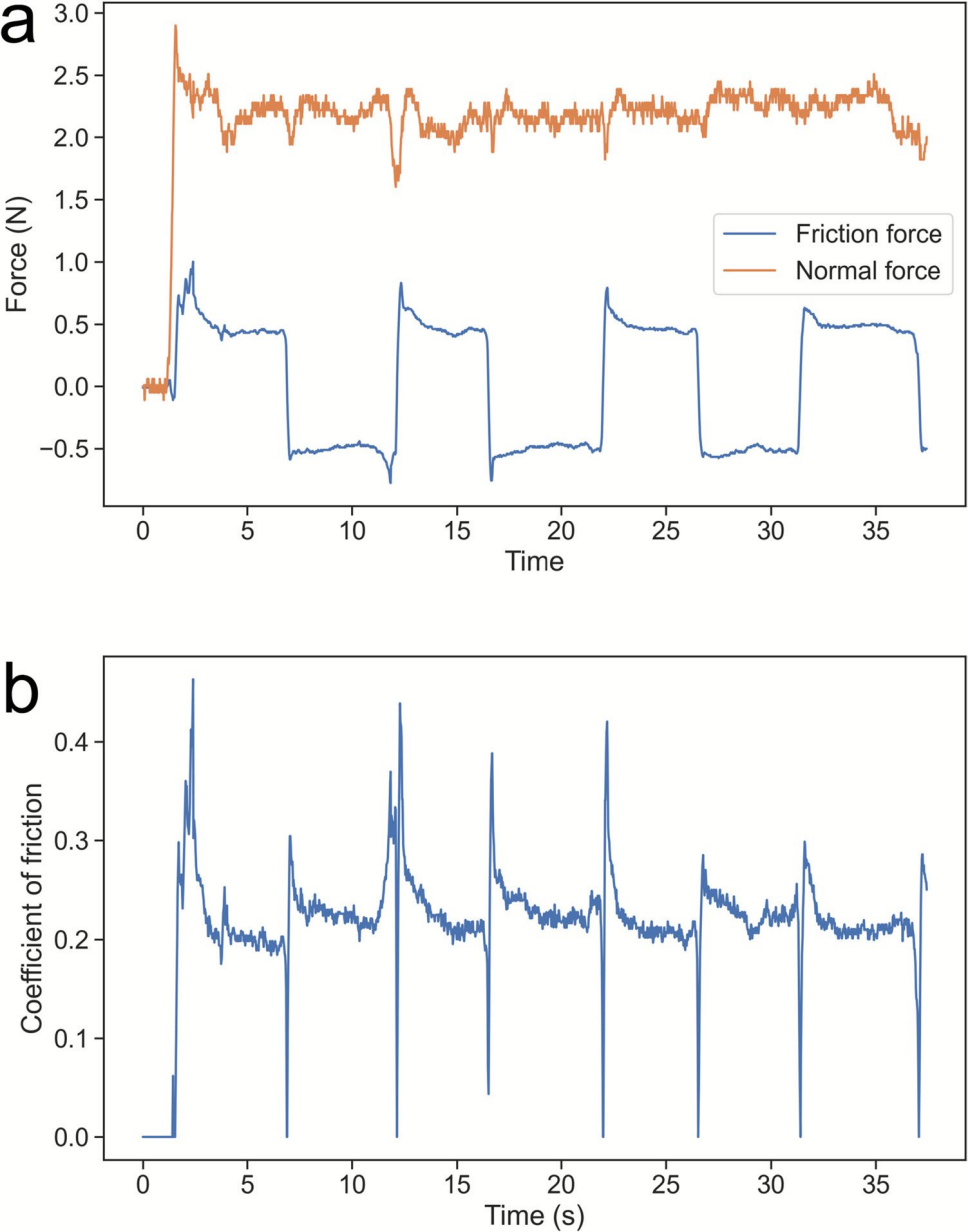
**a** Dynamic evolution of Friction and Normal forces, **b** Resulting Coefficient of Friction—participant A, polycotton knit fabric, 2 N load applied

The raw data (Fig. 4a) shows the evolution of the vertical force applied and the resulting horizontal forces measured upon sliding motion of the finger on the sample. There is a little variation in the normal force applied, which is relatively small considering the nature of the active touch experiments. The horizontal forces measured alternate between positive and negative values, as the sign of the friction forces depends on the sliding direction. The absolute values of the horizontal forces were used to calculate the Coefficient of Friction to obtain positive values of COF in Fig. 4b.

A python code was developed to divide each of the friction profiles into envelopes corresponding to single finger sliding movements to calculate the static and dynamic values of COF. This allows extraction of values of the static COF (95% percentile of the static region) as well as the dynamic COF values (median value of the dynamic region) for each of the sliding movements. The values were computed for each repeat, normal load applied, sample, and experimenter, and used for data visualisation and statistical analysis, such as the Tukey–Kramer comparison test.

#### Acoustic Emission

An acoustic emission sensing setup (Vallen Systeme GmbH, Germany) comprising a passive piezoelectric AE sensor with a broad frequency response in both the standard and high-frequency ranges (Vallen VS900, 100 kHz to 900 kHz, fPeak 350kHz), a preamplifier (Vallen AEP5, 34 dB Gain), and the Vallen AE software suite, was used in the present work to capture the acoustic signal generated by the finger friction processes. A Vallen 2-channel AMSY-6 Data Acquisition Unit (DAQ) was used as a transient recorder. The AE sensor allows for the measurement of the pressure fluctuation associated with the transient elastic sound wave transmitted within the solid substrates upon the friction of the finger on its surface. The AE sensor was placed on an enamel tile with ultrasound gel inbetween to ensure good contact between the sensor and the substrate, and was 2 cm away from the 10 cm long delimited sliding area (see Fig. 3). The other samples were fixed with double-sided tape onto the enamel tile.

**Fig. 5:**
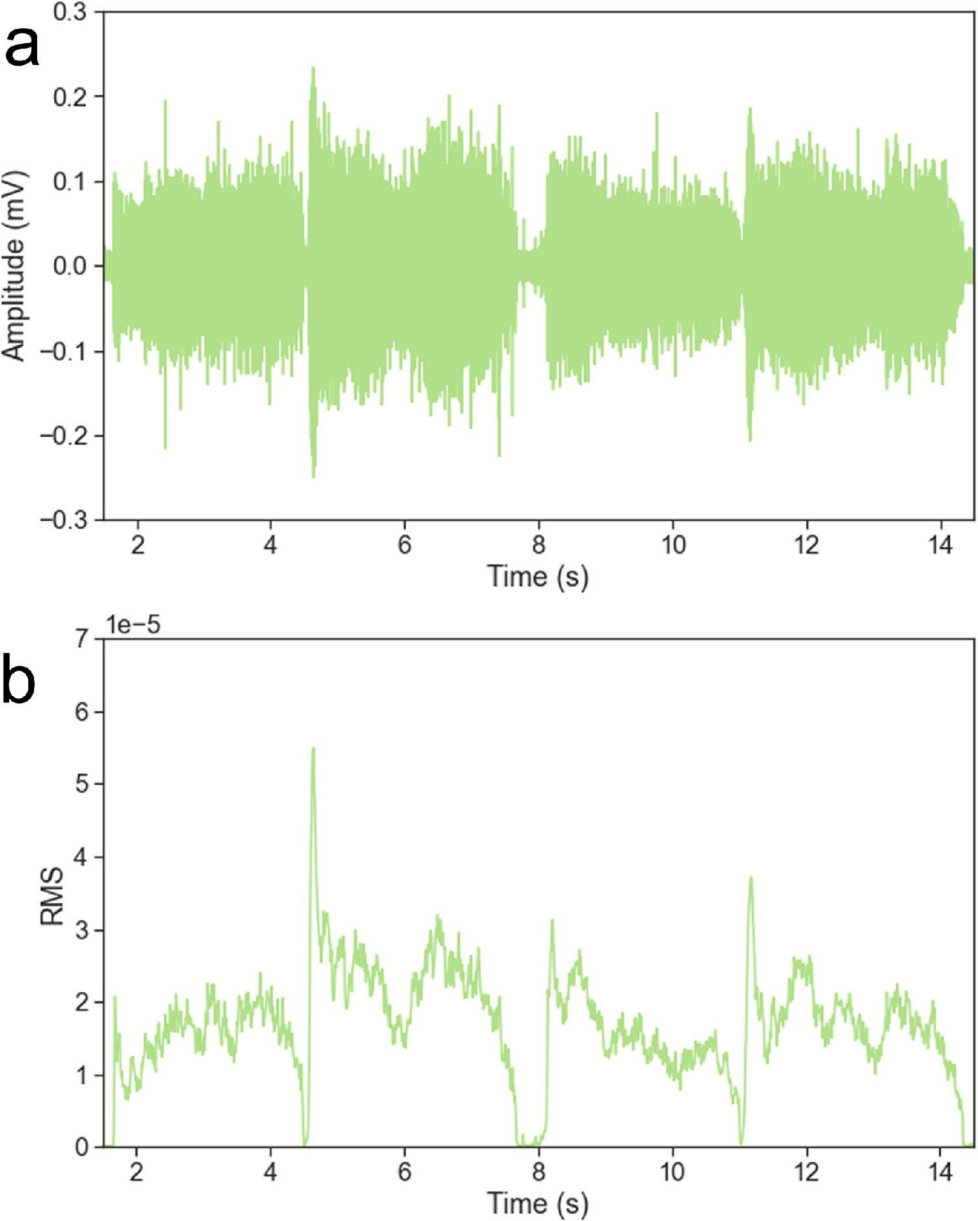
**a** Raw acoustic emission waveform and **b** Root Mean Square (RMS) calculated—participant A, polycotton knit fabric, 2 N load applied

**Fig. 6:**
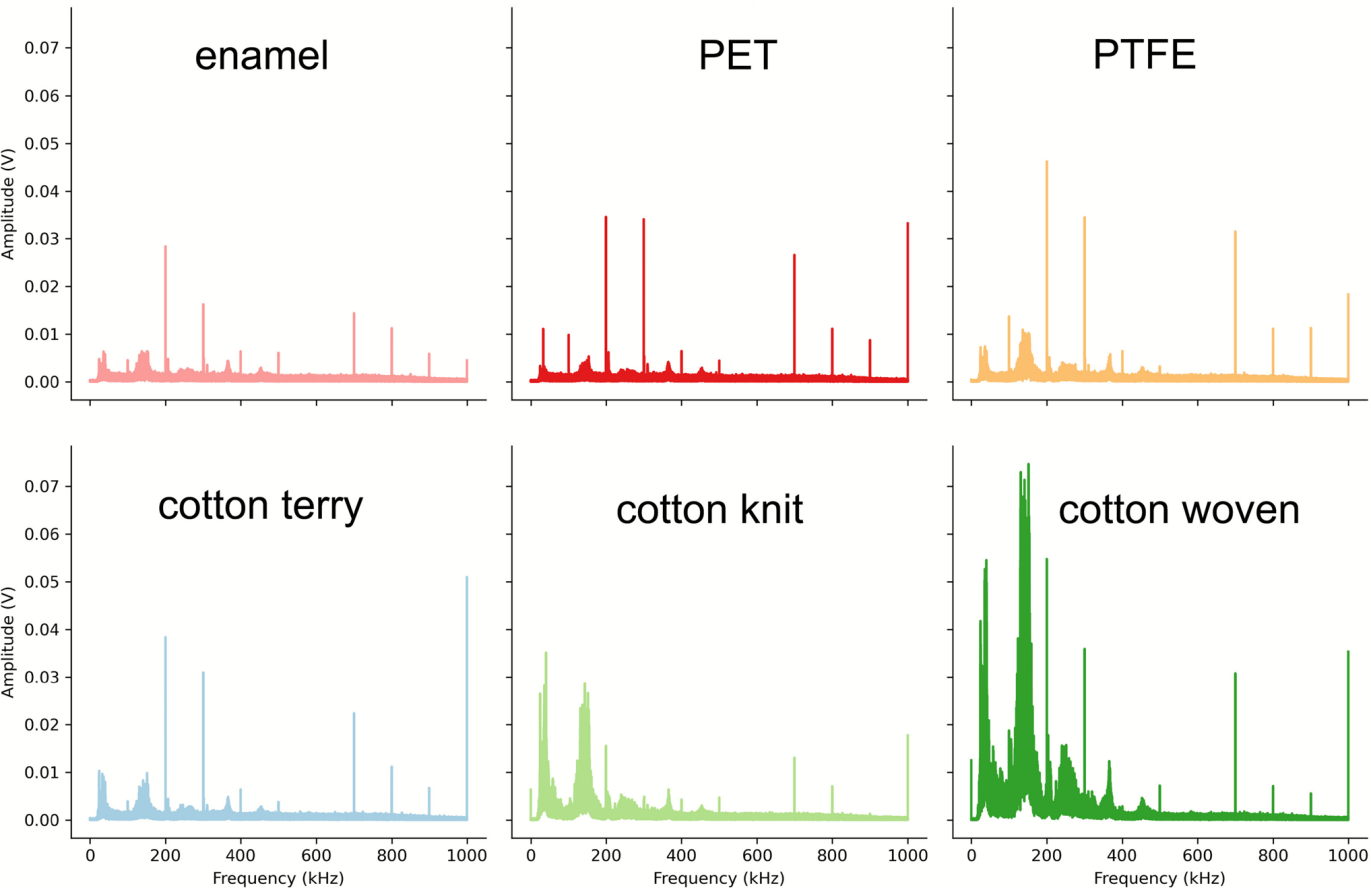
Mean fast Fourier transform (FFT) frequency plots for acquired data on six substrates, enamel, PET, PTFE, cotton terry, cotton knit, and cotton woven, generated by participant A

The raw AE data (Fig. 5a) show a signal waveform in the time domain, with an unsteady behaviour due to the material deformation (intrinsic nature of the AE generation). The time-domain waveforms were processed with Fast Fourier Transform (FFT) to establish the characteristic frequencies of interest (Fig. 6). The frequency-domain plots were smoothed with a customised peak finding function to remove singular peaks (with prominence between 0.01 and 0.1, height above 0.015, and distance of a minimum 50,000 data points) associated with the noise generated due to reversing the travelling direction of the finger. A frequency range of interest was identified for all plots, ranging from 120 to 160 kHz. The top 10% of the data was extracted for each experimental data filtered in this frequency range.

The energy of the AE signal can equally be expressed by calculating the Root Mean Square (RMS) of the raw AE signals. Good correlations between AE RMS and tribological data were previously established [17, 22, 36]. In the present work, RMS was calculated every 20,000 points (0.01 s increments), with the aim of finding a similar correlation for skin in contact with varying materials, using the equation below:

$$xRMS=\sqrt{\frac{\sum_{1}^{n}{x}^{2}}{n}}$$

**Fig. 7:**
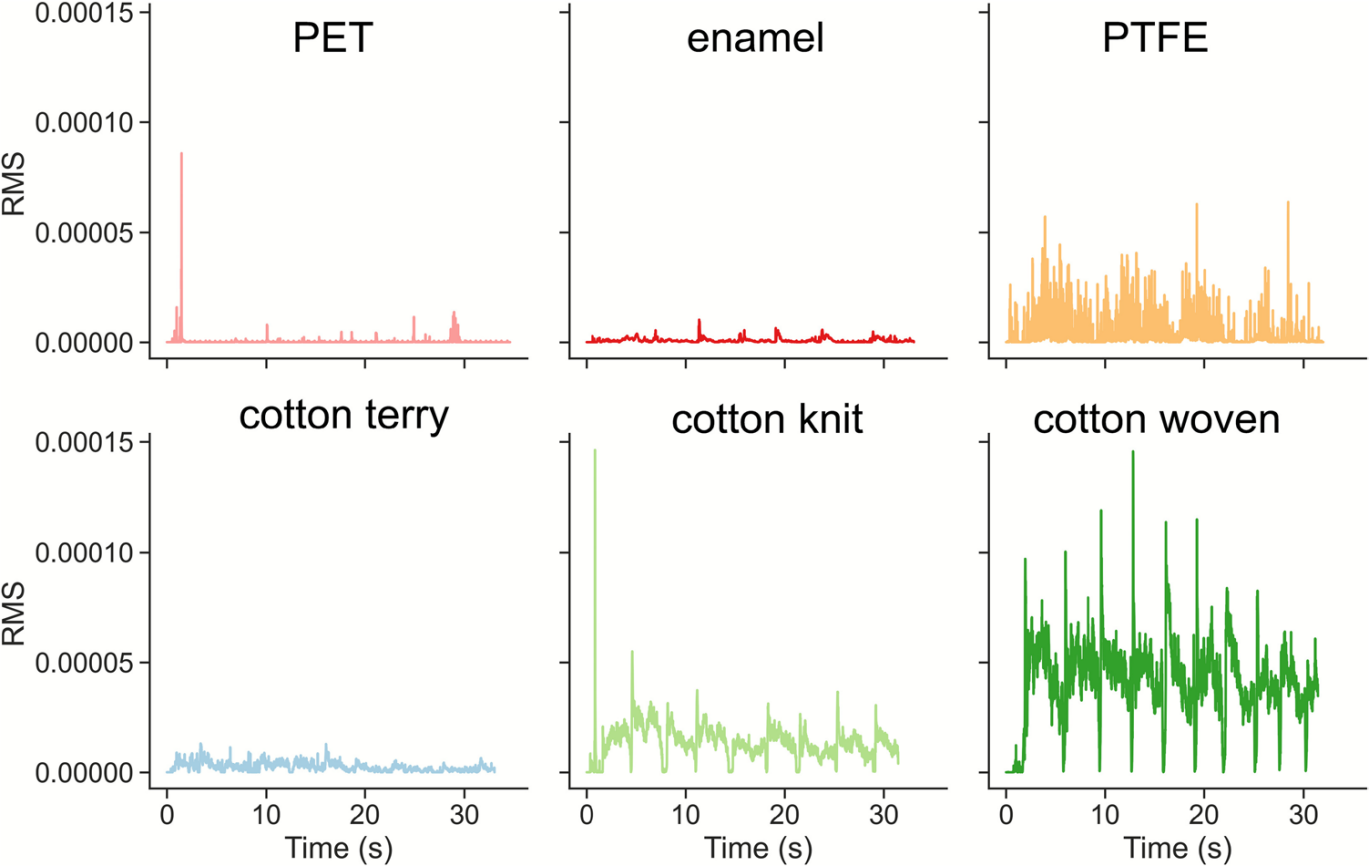
Root Mean Square (RMS) calculated for participant A, 2N applied, for all six materials investigated

Figure 5b shows an example of the RMS profile over time, calculated for the same experimenter and target normal load (Participant A and 2N applied) for the polycotton knit fabric. The RMS profile over time can be assimilated to the friction profile, with both static and dynamic regions identifiable for each sliding movement. The similarity of the AE RMS profiles with the friction profiles was observed for all types of fabric samples investigated (Fig. 7), for each of the participants and target normal loads. This allowed us to perform a further data analysis, in which the sliding movements were separated to extract both static and dynamic RMS values (see Data analysis). However, all non-textile materials showed different RMS profiles, on which the sliding movements or the static or dynamic regions cannot be separated with the same methodology.

## Results and Discussion

### Sensory Questionnaire

Principal Component Analysis (PCA) is a multivariate statistical analysis tool widely used in sensory analysis [37]. It provides a visualisation of a multi-dimensional data set and therefore distinguishes descriptive sensory data. The investigated samples are divided into clusters, and qualitative information on the effect of the different sensory parameters investigated on those samples can easily be obtained visually [8, 38, 39]. Here, we performed a PCA on the sensory questionnaire data from each participant and sensory attribute suggested (stickiness, softness, roughness, thickness, greasiness, and overall feeling (pleasantness)).

**Fig. 8:**
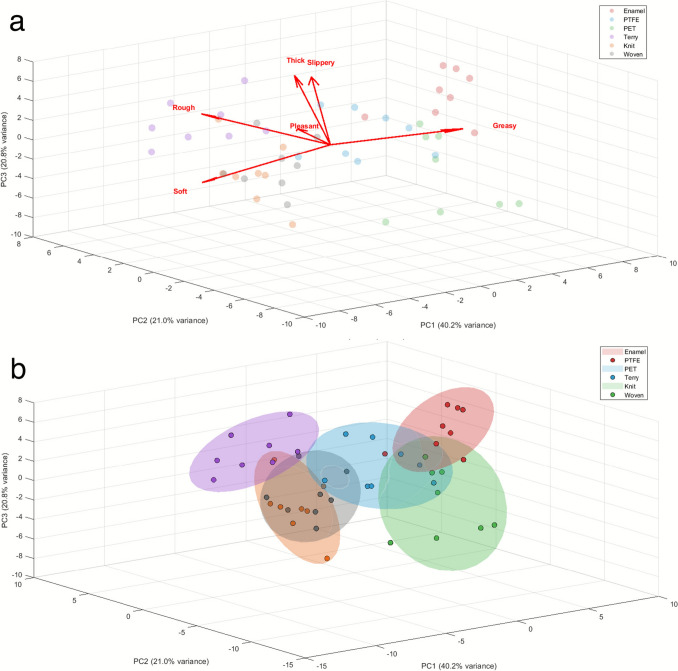
**a** 3D PCA biplots based on the sensory questionnaire data, assigning sensorial benefits with three PCs; **b** 3D PCA scores with material ellipsoids

The first three dimensions of the resulting PCA (Fig. 8) account for 82% of the data. Concentration ellipses show the 95% confidence interval of the distribution around the averaged point (larger data points for each set of data) for each material investigated. It was observed that the flat fabric materials are grouped together, when the terry fabric, having a high roughness associated with the loops pointing out from the surface, was perceived differently by the panellists. The non-fabric anchors have, as expected, sensory benefits different from those of the fabrics. Fabrics, and especially the non-hairy fabrics (knit and woven), were perceived as softer by the panellists than the non-textile materials.

In their review of finger-pad contact mechanics and friction regarding tactile perception, van Kuilenburg et al. identified the distinction between the engineering definition of softness, which is opposed to the indentation hardness (resistance against plastic deformation), and the psycho-physics softness, which corresponds to the compliance or lack of stiffness [40]. It was explained that the perceived softness or hardness of an object impacts which of the elastic modulus or the stiffness would best describe an object’s evaluation of softness.

The sensory parameters investigated (PCA variables) are represented on the plot by the arrows in Fig. 8a, of which the corresponding contribution rates and variable loadings are summarised in Table 1. The quality of each variable is reflected by the length of their respective arrows, with a good representation of the variable for a great distance from the origin of the PCA plot (Fig. 8a). There is no significant difference in the effect of each of the sensory parameters on the principal component analysis. Positively correlated variables are grouped together. Here, slipperiness and thick are highly positively correlated, shown in Fig. 8a—thick, as a perception, tends to relate with lubricated and therefore imply an increased perceived slipperiness. Slipperiness is also positively correlated with the overall pleasantness perceived, suggesting that low skin friction and adhesion would infer the most pleasant tactile perception. Similarly, negatively correlated variables, such as roughness and pleasantness, are positioned in different regions of the plot. In this study, one sample, the terry fabric, can easily be discriminated from the other substrates from a simple visual assessment of the roughness, due to the presence of hairy loops at the surface of this fabric. For the PET film and enamel tile, however, the surface is relatively smooth. The perceived roughness is not necessarily equal to its measured roughness [41], and its sensory evaluation effectively depends on the panellists’ subjective perception of roughness. The roughness perception of a surface not only comprises the topography of the surface but also its friction and compliance [40].

**Table 1:** Variance contribution rates and variable loadings results from the PCA analysis presented in Fig. 8

Variance contribution rates			
PC	Eigenvalue	Variance (%)	Cumulative (%)
PC1	23.4764	40.19	40.19
PC2	12.2912	21.04	61.24
PC3	12.1340	20.77	82.01
PC4	6.1666	10.56	92.57
PC5	2.6346	4.51	97.08
PC6	1.7059	2.92	100.00

Variable loadings			
Attribute	PC1	PC2	PC3
Slippery	0.3692	0.5590	0.3267
Soft	− 0.3365	0.4593	− 0.4970
Rough	− 0.5443	0.2214	0.3890
Thick	− 0.0707	0.1564	0.6656
Greasy	0.5853	− 0.1999	0.0710
Pleasant	0.3265	0.6026	− 0.2164

### Finger Friction

**Fig. 9:**
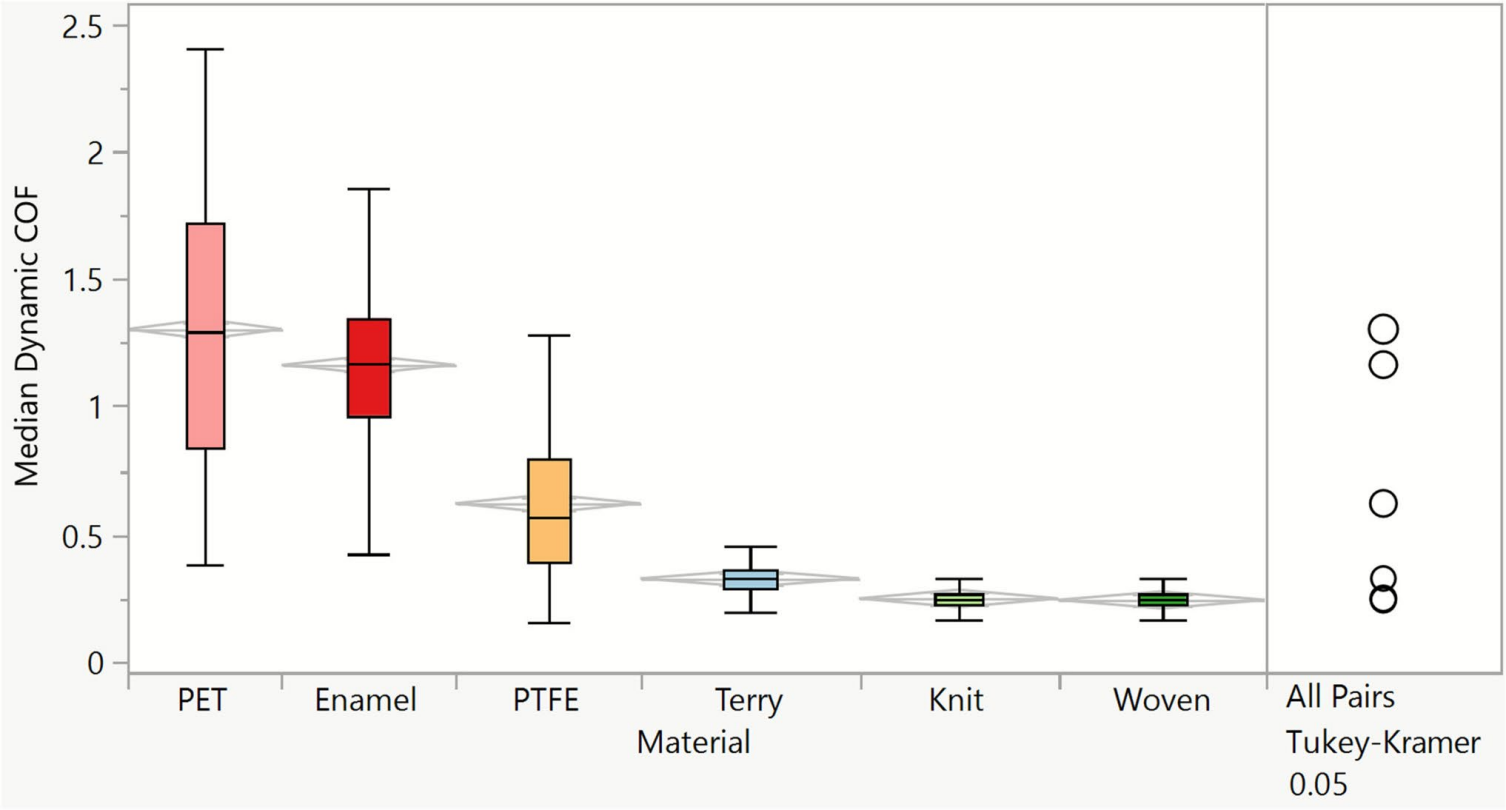
Median dynamic Coefficient of Friction (COF) extracted for all data combined (subjects, target normal load, sliding direction), per material

The dynamic COF of finger friction was calculated for all combined parameters (subjects, target normal load, sliding direction) and plotted as a function of the substrates investigated in a box plot (Fig. 9). Box plots produce a simple distribution visualisation, showing the median value (line dividing the box into two parts) and four sections, from the lower whisker to the upper whisker, each representing 25% of the data.

The reported COF values of skin are commonly between 0.2 and 0.5 but could reach 2 according to the experimental conditions [42], with a representative value for the fingertip found to be 0.81 [43]. In the present work, all COF measured between fingertip skin and the counter substrate (hard or textile substrates) were also found to be within this range, with a few outliers above 2 for contacts between skin and PET film (see Fig. 9).

Figure 9 reveals the considerable difference in the COF values measured for finger friction on the solid substrates (PET film, enamel, PTFE) as opposed to the fabric materials, which all exhibit COF values below 0.5, with a lower amplitude in the values. Similar findings were reported by Fagiani et al. [44], where the values of the measured Coefficients of Friction on fabrics were close to 0.5, and much higher for a non-deformable material such as aluminium. Furthermore, the values of COF are in agreement with those (COF values between 0.45 and 0.75) reported by Gerhardt and colleagues, who measured the friction between the inner forearm and a hospital fabric in the natural skin condition and in different hydration states [45]. In Fig. 9, the Tukey–Kramer pairwise comparison is shown on the right panel, confirming that the COF values obtained for the different materials are statistically different. Here, we found that the hairy structure on cotton terry results in a greater COF than those obtained for the other fabrics, whilst the mean COF values obtained for knit and woven fabrics cannot be statistically differentiated. This highlights the complexity of successfully discriminating fabric types with conventional tribological approaches, with finger friction values of varying types of fabrics in a very similar range. It must be highlighted that our result does not contradict previous work [13] whereby fabrics of varied chemical nature were investigated—it is very likely that the surface energy, governed by the material chemistry, of the fabrics plays a more important role on the interfacial friction than the construct of fabrics of the same materials does.

A greater variation in the COF values was found for hard materials as opposed to textile substrates. The experimenters faced an increased difficulty in applying the target normal load to hard materials, especially on smooth substrates (PET film, enamel). The increased surface contact between the fingertip and smooth samples led to an increased adhesion component of the skin friction, whilst the skin deformation is significant with sliding movement [40]. The friction profiles measured for the sliding movement of the fingers on the smooth materials showed stick and slip phases, which were evidenced by a large variation in friction within the dynamic COF region and corroborated with the sensory feel perceived by the experimenters.

Childs and Henson [46] studied finger friction and sensory perception on polyester sheets with surface roughness from printed inks and reported COF values between 0.63 and 1.1 for similar normal loads applied than in the present study (1.5 N) [5]. The higher COF values obtained against the polyester films in our work could be explained by the smoothness of the PET films and its consequent stick–slip phenomena aforementioned. Masen investigated dry finger friction against steel materials of varying surface roughness and reported an increased COF of the finger with a decreased surface roughness [12]. Skin friction does not follow Amonton’s law, since the increase in normal load applied is not proportional to the increase in friction forces but instead follows the concepts of friction for elastomers for which the Coefficient of Friction depends on both adhesion and deformation components [2, 6, 47]. Fabric samples are highly deformable and show reduced friction coefficients, as opposed to the other samples investigated, which are direction-dependent: mean values from left-to-right and right-to-left motions are statistically different for the textiles investigated, but it is not the case for the non-fabric materials investigated in this work.

### Acoustic Emission

**Fig. 10:**
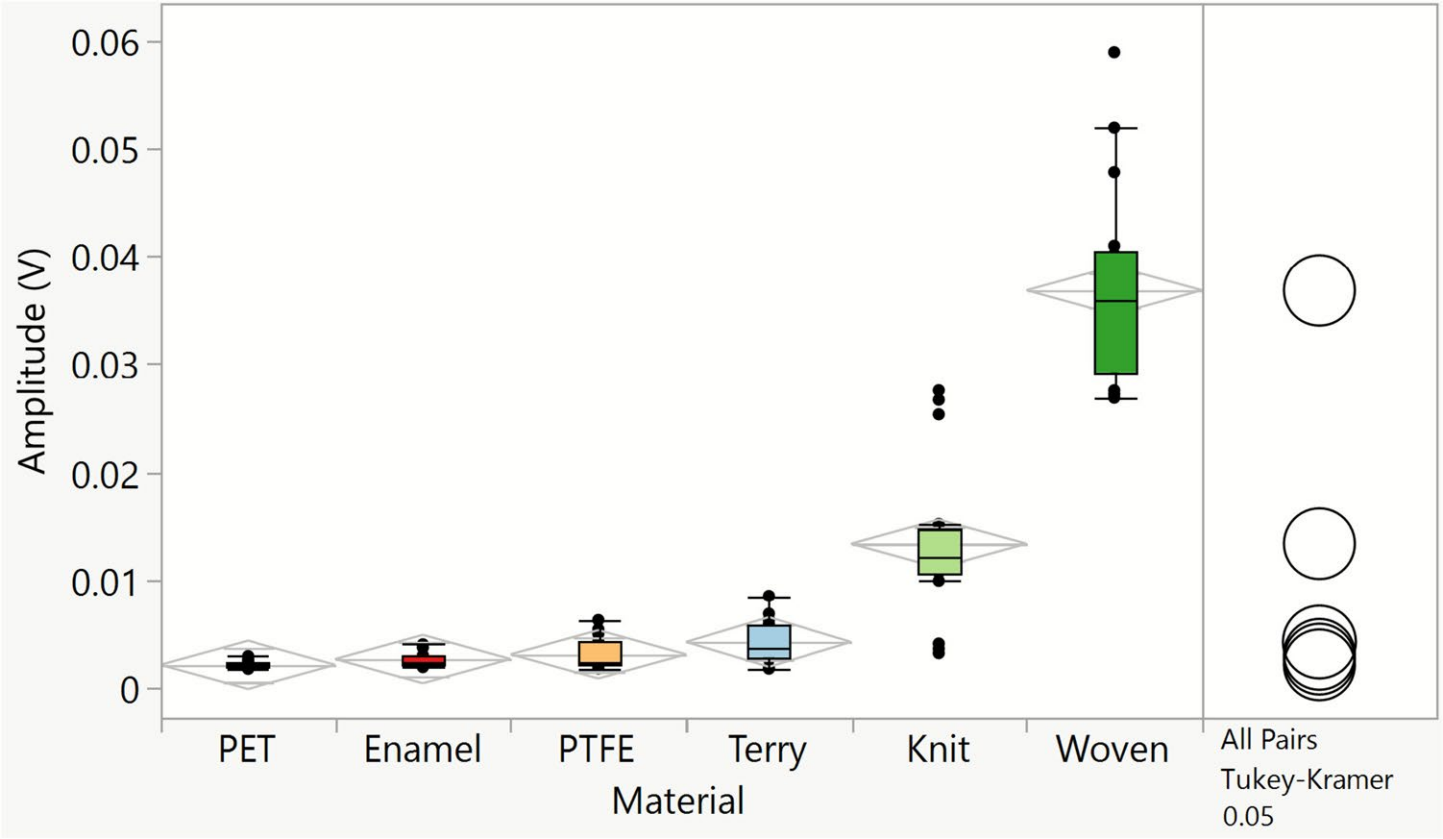
Ninety percent percentile of the acoustic energy extracted between 120 and 160 kHz for all data combined (subjects, target normal load), for all materials

The frequency-domain data within the 120 and 160 kHz region were filtered, from which the 90% percentile was subsequently extracted for each combination of material, participant, and normal load applied. The extraction process allows us to obtain a representative value of the acoustic emission signal magnitude, which is independent of the presence of local maxima in the time-domain acoustic signal. This methodology could also extract energy data from the acoustic signal for the planar materials. The trend obtained by the acoustic emission amplitude values in Fig. 10 is the inverse of the one obtained for friction results, seen in Fig. 9, for all materials.

Another approach to extract data from the acoustic signals was to correlate acoustic emission with the tribological values, based on the similarities found between the tribological and acoustic Root Mean Square (RMS) time-domain profiles. However, since the anchor materials (PET, PTFE, and enamel) did not show a friction-like profile in the time domain, the RMS was solely calculated for raw AE data acquired with the fabric materials. The scope of this study being to investigate fabric materials’ behaviour, it remains interesting to study more in depth the same extraction method for the dynamic and static regions of the friction and acoustic emission RMS time profiles. Moreover, such methodology based on RMS calculation was found to be faster and less computationally intensive than the frequency-domain methodology. For the fabric materials and all combined parameters (subjects, target normal load, sliding direction), static and dynamic AE RMS were calculated using the same calculation method as for the friction coefficients.

**Fig. 11:**
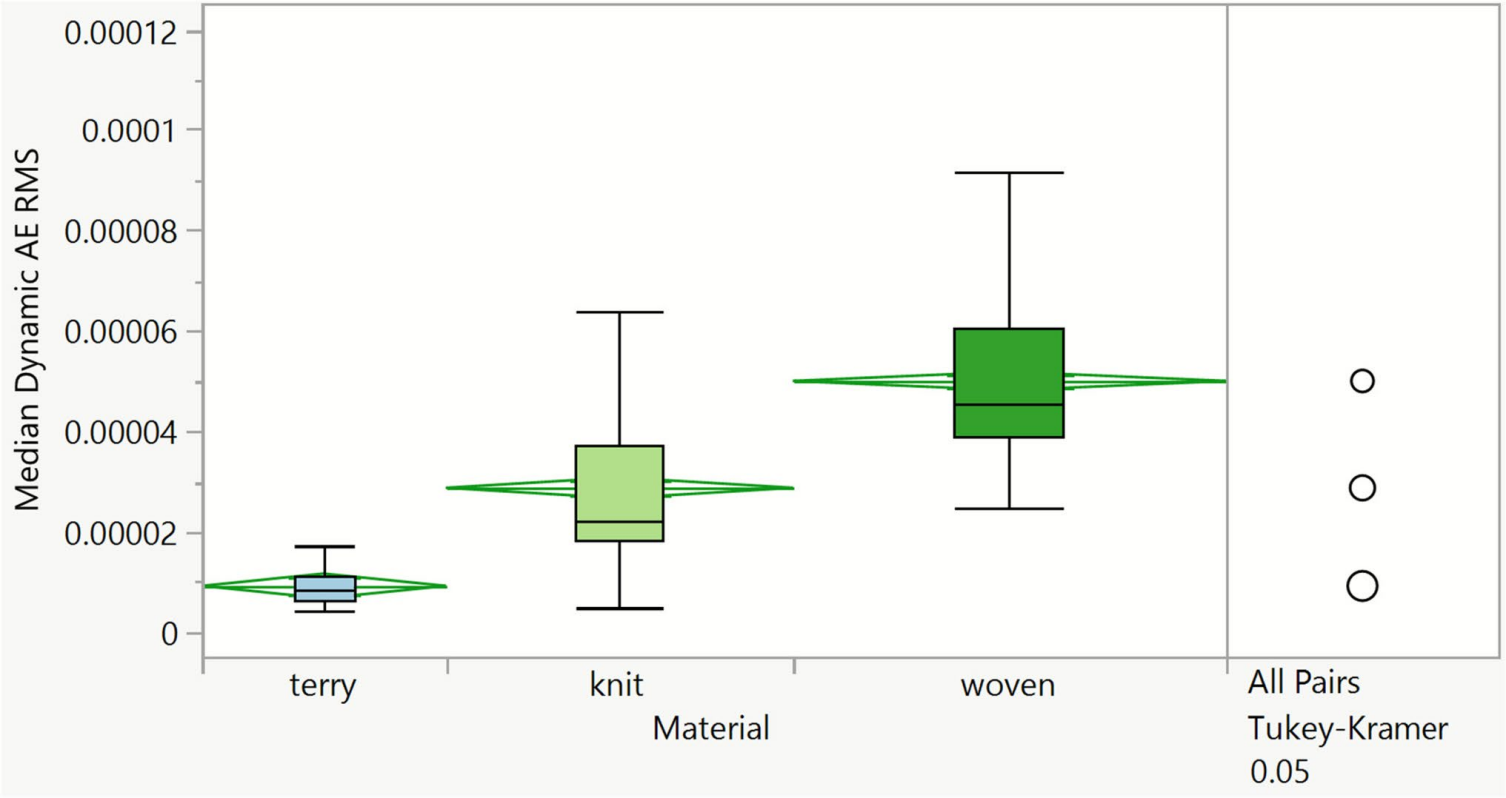
Dynamic acoustic emission Root Mean Squared (AE RMS) extracted for all data combined (subjects, target normal load, sliding direction), per fabric material

The AE RMS box plot of fabric materials (Fig. 11) shows the same trend that was previously established for the 90% percentile of the acoustic signal extracted between 120 and 160 kHz in Fig. 10, with a higher acoustic emission RMS value observed for the cotton woven fabric than for the polycotton knit and with the lowest signal obtained for the cotton terry fabrics. The terry fabric’s AE RMS values are lower with a narrower distribution than for the other fabrics. Acoustic emission measures the energy dissipated by both asperity contact and plastic deformation that occurs with the sliding movement of the finger on the fabrics. The structure of cotton terry fabric comprises numerous surface loops that deform elastically on the surface; therefore, there is a higher energy loss than for the other fabric types investigated in this study. A close correlation of AE signal with conventional tribology measurements such as friction was found for steel-on-steel ball-on-cylinder systems [17, 22]. In soft systems like skin contacting fabric or non-fabric substrates, deformation and sound dissipation have more effect. In this respect, a positive correlation was not obtained in this study. Instead, we show the complementarity of the friction and AE methodologies.

Both AE analysis methodologies presented in this work allow us to statistically discriminate fabrics. This observation suggests that complementary information can indeed be extracted from AE signals, and that friction with fabrics can be successfully investigated with the AE technique. Geng and colleagues also showed that AE signals contain more information than conventional tribological measurements, and that AE is a promising technique to study tribology [22]. In this work, we found that these conclusions are equally applicable for soft tribology systems, such as skin contacts. The RMS-based data analysis approach is more effective for studying fabrics, as this methodology is much faster and less computationally intense than the frequency-based counterpart, giving an excellent correlation. It is worth highlighting that RMS is a time-domain feature that was used to visualise and compute the tribological processes that are dynamic in nature, whilst the Fourier transform is used to show the characteristic response from the material interaction (event location), which is unable to capture the dynamic changes. Admittedly, the application of the FFT is not ideal, given it assumes a steady state; however, it is useful to summarise in a concise manner where events happen and what the nature of these events are (energy levels).

### Correlation of Sensory and Tribology

**Fig. 12:**
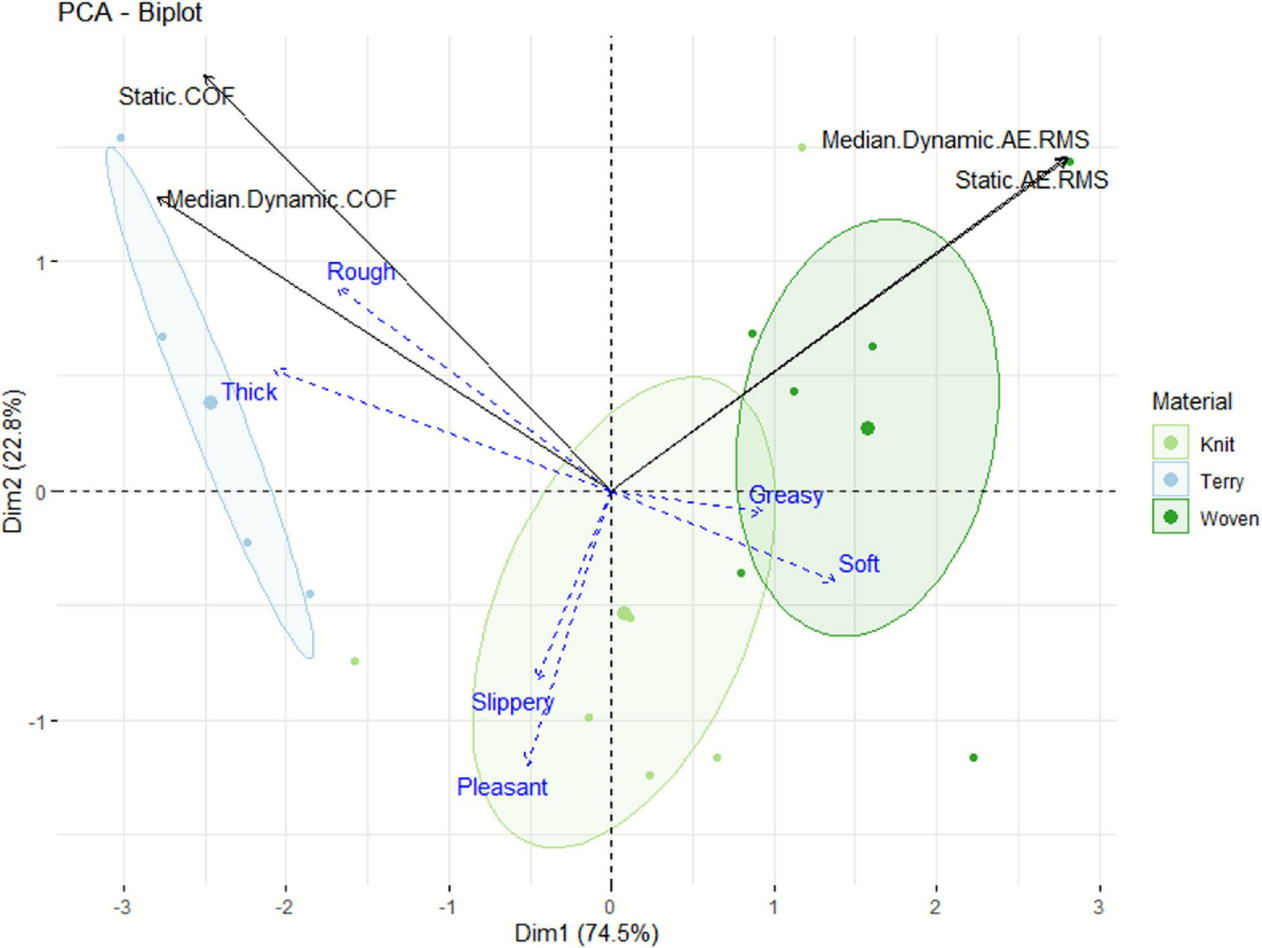
Principal component analysis of the mean static and dynamic COF and AE RMS values obtained for each fabric material, per participant. Blue vectors represent sensory evaluations

A PCA biplot was generated from the static and dynamic values of both COF and AE RMS as main variables (Fig. 12), alongside the sensory data. From this analysis, we observed that the Coefficient of Friction is positively correlated with perceived roughness, and negatively correlated with perceived softness of fabrics. A lower friction coefficient is usually preferred for an enhanced perceived fabric softness, and more generally for a preferred tactile feel [9, 48, 49]. Roughness is intrinsically correlated with the Coefficients of Friction obtained, since the real contact area is much smaller for a coarse surface than for a smooth surface [2, 50]. Skedung et al. reported that the limit of human tactile discrimination for surface roughness and wrinkles was in the nanometre range [51]. The RMS of AE data is negatively correlated with the perceived slipperiness and overall pleasantness. The acoustic energy emitted is lower when a surface is slippery, as there is less deformation and less energy dissipated. Pleasantness is a difficult parameter to interpret since it relies on multiple sensory parameters, and its very own definition is even more dependent on the individual performing the evaluation than for a single sensory parameter to evaluate. This observation illustrates how the use of AE in the finger friction measurements can offer complementary information on the tactile perception of the fabric samples investigated.

## Conclusion

In the present work, we developed a new methodology that combines a tribological rig (force-plate apparatus) with an AE sensor to investigate the finger friction on solid substrates of varying characteristics (textile and planar materials), aiming to correlate the results of physical parameters with sensory response. Tribological characteristics, Coefficient of Friction specifically, were found able to differentiate solid materials but not fabric materials. This highlights the complexity of skin friction that cannot be interpreted by mechanical signals only. The simultaneous measurement of the acoustic signal allows a better understanding of the tribological properties of the different fabric substrates investigated in this study, by providing additional information that allows effective differentiation of fabric materials. Although drawing conclusions from raw sensory data proved difficult, the use of Principal Component Analysis showed promise in identifying the role of both measuring methods in explaining specific sensory attributes. For example, perceived pleasantness and slipperiness of fabric materials were found to be inversely correlated with acoustic emission RMS values. Therefore, low AE values are preferred for an easier sliding motion, which implies tactile pleasantness. Tactile perception is complex since it relies on both tribological parameters and subjective evaluation from individuals; therefore, the use of a complementary technique to study skin tribology, such as acoustic emission, can help to design a model to predict tactile perception of some materials, and especially for fabrics. It must be noted that there are limitations to the tactile perception interpretation in the present work, e.g. the number of panellists recruited is relatively low, which influences the quality of the correlation between the physical measurements (CoF and AE) and the perception. Furthermore, untrained panellists were used, which may have introduced bias in evaluating subtle tactile differences. Both factors should be considered in establishing a comprehensive relationship between the physical and psychological aspects of tactile perception, although it does not undermine the potential of the method reported in the present work. Lastly, we would like to emphasise that a typical value, 0.05, was used as the significance level (*α*) in the Tukey–Kramer analysis, which implies that there is a 5% chance of any pair of means being declared significantly different when they are actually the same, across all comparisons. This could be improved with an increased number of panellists for future work.

## Data Availability

All data supporting the findings of this study are available within the paper.
